# The Meaning of Physical Activity: A Qualitative Study on the Perspective of South American Immigrant Parents

**DOI:** 10.3390/ijerph17207500

**Published:** 2020-10-15

**Authors:** Romain Marconnot, Antonio Luis Marín-Rojas, Carmen Jiménez-Antona, Jorge Pérez-Corrales, Javier Güeita-Rodríguez, Domingo Palacios-Ceña

**Affiliations:** 1 Área de Educación Física del Departamento de Fisioterapia, Terapia Ocupacional, Rehabilitación y Medicina Física, Universidad Rey Juan Carlos, Avenida Atenas s/n, 28922 Alcorcón, Spain; romain.marconnot@urjc.es (R.M.); antonio.marin@urjc.es (A.L.M.-R.); domingo.palacios@urjc.es (D.P.-C.); 2 Departamento de Fisioterapia, Terapia Ocupacional, Rehabilitación y Medicina Física, Universidad Rey Juan Carlos, Avenida Atenas s/n, 28922 Alcorcón, Spain; carmen.jimenez@urjc.es (C.J.-A.); jorge.perez@urjc.es (J.P.-C.); 3 Research Group of Humanities and Qualitative Research in Health Science (Hum & QRinHS), Rey Juan Carlos University, Avenida Atenas s/n, 28922 Alcorcón, Spain

**Keywords:** immigrants, physical education and training, exercise, youth, parents, qualitative research

## Abstract

Physical activity facilitates the acquisition of healthy habits from childhood to adulthood. There are differences in the practice of physical activity between immigrant children and native Spanish children. The aim was to describe physical activity in children, from the perspective of South American immigrant parents. A qualitative case study was conducted. Parents were recruited using purposeful sampling. Data were collected from 12 participants by means of unstructured interviews together with researcher notes. A thematic analysis was applied. The following topics were identified: (a) physical activity and health, (b) socialization, (c) the need for movement, (d) gender, (e) barriers, (f) educational burden, (g) community living, and (h) reason for immigrating. Parents described how physical activity is fundamental and has benefits for health, and for the relationship between children. They perceived that physical activity should not be limited by gender. The time dedicated to other school activities, the norms related to community living, and financial limitations were especially relevant as barriers for the performance of physical activity. These results can be used to revise the curriculum in schools, promote equal opportunities for physical activity and support family participation.

## 1. Introduction

The World Health Organization (WHO) [1] states that physical inactivity is the fourth leading risk factor for mortality worldwide (6% of deaths) after hypertension (13%), tobacco use (9%), excess glucose (6%), ahead of obesity (5%). The WHO [1] recommends 150 min per week of moderate aerobic physical activity or 75 min per week of vigorous aerobic physical activity or a combination of both. In Spain, the National Health Survey in Spain in 2017 [2] describes how 36.04% of the interviewees perceive themselves to be sedentary in their free time. This figure varies according to nationality, with 35.02% of individuals born in Spain admitting to being sedentary compared to 43.18% of individuals whose country of origin was not Spanish (immigrants). This difference increased in individuals aged between 15 and 24 years old by more than 10 points, and by 12 points in individuals aged between 25 and 64 years old. Immigrant men and women are more sedentary compared to Spanish men and women in the same age groups. However, minors of foreign nationality are more active in their free time than natives (up to six points difference among sexes).

Previous studies [3,4,5,6,7] conducted in the USA and European countries show that children with foreign parents were less physically active than children with native parents. Griffiths et al. [6] described how, in children over the age of seven with immigrant parents, 40% had a level of physical activity considered “adequate” compared to 50% of children with native British parents. Recent European studies show that young immigrants have more difficulty accessing sports clubs and are less physically active than natives [8,9,10]. However, the study by Reimers et al. shows that foreigners participate much more in the extracurricular activities offered at educational centers [8]. The difference in the manner in which native people and foreigners practice physical activity can change when immigrants have been in the country longer or if they are second-generation immigrants [11,12]. This difference decreases if the children are second-generation immigrants [4].

Physical activity in children and adolescents becomes important in relation to obesity. Obesity is a non-transmittable disease considered by the WHO as the fifth mortality factor in the world [1]. Obesity in children and adolescents is a very important factor in early metabolic syndrome and the development of cardiorespiratory diseases [13]. It is a major health risk, although it can be overcome through physical activity [14]. In Spain, according to the National Health Survey [2], Spaniards show a higher percentage of overweight and/or obesity than the immigrant population. However, between 18 and 24 years of age, the immigrant population is more overweight and/or obese than the native population (32.95% versus 24.09% respectively). Foreign women, present greater overweight and obesity between 18 and 64 years of age, compared with Spanish women. In the case of children, there are higher rates of obesity among Spanish children than in immigrant children, in both sexes. These findings are in contrast with a report by Marín-Guerrero et al. [15] in the Community of Madrid who reported a high prevalence of obesity among the foreign population. A review published in 2014 [16] also demonstrated this prevalence of obesity among the immigrant population.

Other factors, such as environmental and/or socio-ecological factors influence the practice of physical activity or the prevalence of obesity [17,18,19,20]. Previous qualitative studies [7,11,21] reported that the factors that limit the practice of physical activity among immigrant children are both environmental barriers (insecurity, lack of sports or leisure resources, and lack of transportation), and socioeconomic barriers (lack of time or income), as well as cultural barriers (religion/beliefs, educational level, language, or gender inequality), which may prevent or limit the practice of physical activity among immigrant children, compared to native children. In addition, the parents’ sporting and eating behavior are also relevant factors in children’s behavior [14,22,23], as they act as role models. Martínez-Andrés et al. [24] describe how parents organize family life according to their own perspectives and priorities, putting educational activities first and leaving physical leisure activities for later.

In Spain, the immigrant population represents 10% of the total population in 2019 [25], and it is predicted that the immigrant population will represent up to 20% of the total population of Spain in 2033 [26]. This increase in population means that new families will be incorporated, with habits and customs, which could be subject to risk and/or protective factors such as physical activity or sedentary behavior. At this point, the authors believe that the parents’ perspective is fundamental in order to establish adherence to healthy habits such as physical activity.

The questions guiding this study are: How do immigrant parents perceive physical activity? What does it mean to them? Do they consider that exercise to be an educational asset or a healthy lifestyle habit that is passed on to their children? Answering these questions may help to understand the role of parents in the practice of physical activity among immigrant children and adolescents and in relation to the acquisition of healthy habits. The aim of this study was to describe the perspectives of immigrant parents on physical activity and how this applies to their children.

## 2. Materials and Methods

### 2.1. Design

A qualitative exploratory case study, using an interpretivist paradigm [27]. A case study is a research design that involves an examination of a contemporary phenomenon in a real-life setting [28]. It is used to explore and describe a single case bounded in time and place (i.e., the parents’ perspective regarding their children’s physical activity). Moreover, a qualitative case study can be used to describe participants’ experiences regarding care, policy change, and understand why behaviors and complex interventions succeed or fail [28]. This research followed the international recommendations for conducting qualitative studies established by the Consolidated Criteria for Reporting Qualitative Research (COREQ) [29] and the Standards for Reporting Qualitative Research (SRQR) [30] (http://www.equator-network.org/).

### 2.2. Study Context

Alcorcón is a municipality of 33.73 km^2^, located 13 km southwest from Madrid, in the Metropolitan Area of Madrid, within the province of Madrid (Community of Madrid, Spain). The greatest population growth took place in the 1970s, mainly due to the settlement of immigrants from other regions of Spain [31]. Currently, in the Community of Madrid, where the municipality of Alcorcón is located, the percentage of physical inactivity among adolescents is 30%. In relation to the situation of the immigrant population in terms of physical activity, the Community of Madrid reports a percentage of inactivity of approximately 39% among immigrant adolescents [2]. Alcorcón is one of the municipalities with the largest foreign population in the community of Madrid, and therefore there is a need for studies to deepen our knowledge of the physical activity dynamics among this group.

For this reason, the authors decided to visit institutions or centers where the population of South American immigrants gather for worship, or for cultural and civic activities, and where there were greater possibilities of recruiting participants for the study. The Caritas organization of the Parish Inmaculada Concepción de Nuestra Señora and the Parish Nuestra Señora de la Saleta, where large groups of South American immigrants met every Sunday and holiday, were particularly relevant.

The participants attended the Parish Inmaculada Concepción de Nuestra Señora (Alcorcón, Madrid) and the Parish Nuestra Señora de la Saleta (Alcorcón, Madrid). Both these parishes had programs for assisting and supporting the integration of immigrant families. These programs included the implementation of healthy habits in children and adolescents through physical activity.

### 2.3. Participants

The inclusion criteria were; (a) parents living in the Community of Madrid, (b) born in countries outside Spain and the European Union, (c) who at the time of the study had children over three years old in the Spanish education system, (d) who belonged to the Caritas organization of the Parish Inmaculada Concepción de Nuestra Señora or the Parish Nuestra Señora de la Saleta, and (e) who signed the informed consent. The exclusion criteria were: (a) parents under 18 years of age, (b) refusal to participate in the study, (c) native parents (born in Spain) and (d) not signing the informed consent.

### 2.4. Sampling Strategy

In qualitative research, sampling is the process of selecting or searching for situations, contexts and/or participants that provide rich data of the phenomenon of interest [32]. Non-probabilistic purposive sampling was performed [33], in which participants were selected based on their ability to provide relevant information to answer the study question. Sampling continued until the ongoing analysis revealed data redundancy [32]. Finally, 12 participants were included in the sample and none withdrew from the study.

### 2.5. Recruitment

The participants were contacted through the priest and coordinator of the family care program. Potential participants (parents) were then contacted by telephone and informed of the study. After a two-week period, they were contacted once again to confirm their participation.

### 2.6. Data Collection

Data were collected via unstructured interviews conducted in Spanish [33]. These interviews allow for inquiry and questioning in an open and flexible manner without imposing the researcher’s perspective [34]. This enables participants to describe and narrate what is most relevant to them. The interview consisted of several open-ended questions: How is your experience with physical activity? Do you think it is relevant for your children? Based on these questions, the researcher wrote down the words, concepts and expressions that the participants used to cross-examine them. Repeating the cycle over and over again, deepening the participant’s experience [33]. Overall, 20 interviews were conducted, and 20 researchers´ field notes were collected. Eight participants were interviewed on two separate occasions, as it was necessary to collect more information. The remaining participants received a single interview.

### 2.7. Data Analysis

A thematic analysis was applied for the description and interpretation of parents’ narratives [35]. The analysis was conducted on all interview transcripts [34]. In the coding process, the descriptive components that made up the participants’ experience were identified, these descriptive components formed meaning units, which were then grouped into common groups of meaning, to finally identify the themes that described the participants’ perspective [35].

### 2.8. Ethical Considerations

The study was approved by the Ethics and Clinical Research Committee of the Rey Juan Carlos University (code: 3001201702417). In addition, the Data Protection Law (Law 15/1999) was complied with, guaranteeing confidentiality and good practice standards. Participation in the study was voluntary and informed consent was signed prior to conducting the interviews.

### 2.9. Quality Criteria

In addition to following the COREQ [29] and SRQR [30] recommendations, the Guba and Lincoln criteria were applied to establish credibility, transferability, dependability, and confirmability [36]. The techniques to ensure credibility were; researcher triangulation, triangulation of data collection instruments, and participant validation. For transferability, the research protocol, and the context and participants of the study were described in detail. An external auditor/researcher was used to ensure confidence. This external auditor reviewed the research protocol, focusing on the development of materials and methods. External audits involve having a researcher who is not involved in the research process examine both the process and product of the research study. The purpose is to evaluate the accuracy and evaluate whether or not the findings, interpretations and conclusions are supported by the data. In the present study, four external audit trials were performed, prior to commencing the study (before February 2017), twice during the study (from February 2017 to June 2017; in March and May 2017), and once after completion of the study (after June 2017). Finally, for the purpose of confirmability, the researchers’ reflexivity process was applied, holding meetings to establish the research team’s prior position on the topic under study [33].

## 3. Results

The study began in February 2017 and was completed in June 2017. Twelve participants were included (seven women and five men), with a mean age of 41. The participants came from different regions of South America (Argentina, Colombia, Ecuador and Honduras). All were defined as having a low or low/middle socio-economic level. The demographic characteristics and physical activity habits of participants are shown in Table 1.

**Figure 1:**
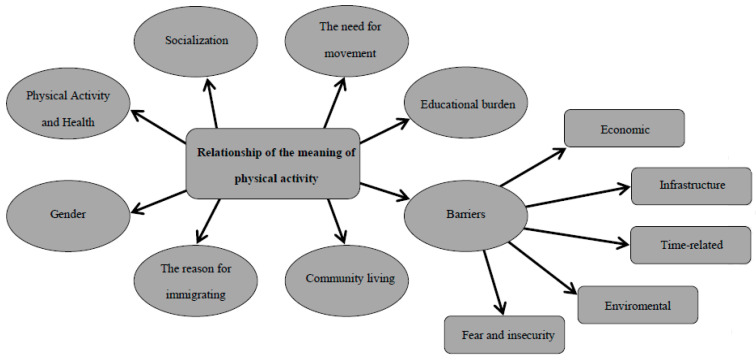
Conceptual map of results.

The themes identified were: Physical Activity and Health, Socialization, The Need for Movement, Gender, Barriers, Educational Burden, Community Living and The Reason for Immigrating. The results are presented below with fragments of the participants’ original narratives to justify the results obtained [33]. Additionally, Figure 1 shows our findings using a conceptual map [37].

### 3.1. Physical Activity and Health

Participants (*n* = 9) described how physical activity leads to better fitness, better health, improved quality of life and physical well-being: “Being more active, more agile, and breathing better, feeling better in general” (P5, female, income level IV).

In addition, they also referred to how physical activity improves mental health: “You have to exercise to be well, to improve your self-esteem, in terms of mental health” (P1, female, income level V); “Changing your routine, obtaining relief, is a way of relieving everyday tension, even, calming the mind” (P4, male, income level IV).

### 3.2. Socialization

For parents (*n* = 8), physical activity supports socialization and helps instill certain values in their children. The narratives highlight the relevance of this for parents: “It is very important for them to practice physical activity, to socialize and get along in life” (P2, male, income level V); “They share with friends at school and learn to share with people outside school, in the parks” (P4, male, income level IV); “Doing something in a group is important. Belonging to a team is important” (P5, female, income level IV).

The narratives (*n* = 5) highlight the belief that practicing physical activity as a child can help their children be healthier when they reach adulthood: “You will always have better health conditions if you move, than if you are a sedentary person” (P6, male, income level III).

### 3.3. The Need for Movement

For the interviewees (*n* = 5), movement was a recurrent theme when qualifying and defining physical activity. Physical activity consists of movement, agility, physical space, the natural environment and play. In contrast to being sedentary, or confining a child to the house, which was compared to being cooped up in a cage: “Children live in cages, they are locked up” (P9, female, income level III).

Seven participants also compared the way children play and practice physical activity in Spain in relation to their countries of origin. Thus, in Spain, both children and people, in general, spend more time indoors, and consequently, they are more sedentary. In addition, they voiced their concerns with regard school environments, feeling that movement should be granted equal or greater importance as intellectual activity: “They need to get out of the chair and not just exercise the brain, which is also important” (P12, female, income level V).

### 3.4. Gender

The narratives also revealed differences in sports practice by gender. All participants unanimously stated that there should not be any difference in physical activity practice between boys and girls. Thus, no activity should be reserved for only one sex. Every child should be free to choose and practice the activities he or she desires: “I don’t think that sport has anything to do with sexual personality. As long as they feel good” (P10, male, income level V).

However, all parents’ acknowledged differences between boys and girls. Boys play more football and girls play more sports like volleyball or gymnastics: “Football is played by boys. There are more and more girls, but it’s a boy thing. In my country, girls practice a lot of volleyball and rhythmic gymnastics. It is very rare for boys to play such sports” (P6, female, income level III).

### 3.5. Barriers

Barriers encountered by parents when encouraging and/or practicing physical activity with their children include: economic barriers, infrastructure barriers, time-related barriers, environmental barriers, or barriers due to fear and insecurity.

#### 3.5.1. Economic Barriers

Eight participants reported that families must cover significant economic expenses when their children engage in any kind of organized or regulated physical activity: “If you want your child to practice, you have to pay and that seems overwhelming to me” (P1, female, income level V); “If you don’t have help, I can’t afford it. It’s an expense I can’t afford.” (P2, male, income level V).

A difference was highlighted between the country of origin and Spain. Family benefits are more extensive in Latin American countries, allowing families to participate in physical activities with their children: “There are associations that provide free training. There are also sports centers belonging to the city council, and everything is free” (P8, male, income level V).

The concept of “sacrifice” appeared in the participants’ narratives (*n* = 5). Families much make a great sacrifice in order to enable their children to practice some kind of activity: “I know many friends who have to make great efforts to get their children to play soccer, they sacrifice a lot for them” (P6, female, income level III).

Finally, having to register with a club, or a sports school, is considered another barrier for four participants. In addition to having to pay registration fees, specific material equipment is required: “Here you must pay for everything, you have to put them in a defined, organized team. If you don’t have the right boots, you can’t play” (P11, male, income level IV).

#### 3.5.2. Infrastructure Barriers

Our participants (*n* = 7) reported that the availability of sports and/or leisure infrastructures near the family home, and their distance influences the child’s physical activity. The narratives show that the absence of facilities conditions and prevents children’s engagement in physical activity: “In the case of Parla, it is an abandoned village. There is no place to do sport. There is neither a field nor a sports center to take them to, there are no sports areas” (P1, female, income level V).

Parents (*n* = 5) reported that the greater the distance from the facilities, the less likely their children are to practice physical or sporting activities. Substantial time is involved in travelling to and from these centers: “There is a lake, it is a very big park, but mothers do not always have the availability to go there every day. It’s far away and, without a car, it’s impossible” (P9, female, income level III).

#### 3.5.3. Time-Related Barriers

In order for their children to be physically active, parents (*n* = 7) must invest large amounts of their time. However, often, parents (*n* = 4) complained of a lack of time available, either because of an extended working day, and/or because their work hours are incompatible with the child’s leisure time. Six parents described that their working hours are intense and this does not allow them to take their children to organized physical activities or to the park: “Work has a great influence, you cannot spend enough time with them (the children) either. If you don’t have enough time, you can’t do it either” (P10, male, income level V).

This lack of time influences children’s physical activity, as well as having an impact on parents’ practice (*n* = 5). Thus, parents assigned priority to their children’s practice of physical activity and leisure above over their own: “We have a lot of work. And I prefer to spend more time with my children instead of going to do sport” (P4, male, income level IV); “But I can’t do sport with the children anymore. For me it is a big obstacle to do physical activities” (P12, female, income level V).

Other key points raised were: the lack of time, organizing the child’s schedule and the pace of the day. The participants (*n* = 3) described how the proposed timetable for practicing sports activities was unsuitable, both for their children and for the ability to balance work/family life: “The extra-curricular activities that we can take them to, which are often more compatible with my work, are more expensive” (P5, female, income level IV); “My son had a scholarship because he came first at a sports meet at the town hall, but the timetable was from 6.30 p.m. to 7.30 p.m., and I can’t take them at that time. What did I end up doing? We took him out, we couldn’t go” (P8, female, income level V).

#### 3.5.4. Environmental Barrier

The parent´s narratives (*n* = 5) describe how the lack of nature or a natural environment near the homes is a constraint for engaging in physical activity: “In my country, there is more freedom to play sports outdoors than here, the children still go down to the street to play football” (P2, male, income level V); “We are much more limited here by the surrounding environment” (P7, male, income level IV).

#### 3.5.5. Barriers due to Fear and Insecurity

This refers to the insecurity and fear that parents (*n* = 7) feel when they take their children to physical activities in an unsafe environment. Some (*n* = 3) felt “terrified” due to the feelings of insecurity when going to some of the parks. In addition, if there are areas that are not recommended for children because of insecurity, they feel that it is unsafe to leave children alone in the street to play ball: “The courts are full of people who hurt the children, and as a mother I feel afraid to let them go” (P2, male, income level V); “Here you feel terrified of taking them. There’s a big park, but since everyone goes, it scares you. And in the street you can’t leave them alone either” (P11, male, income level IV).

In contrast, participant 3 expressed opposite feelings, in Spain he did not fear taking his children to the park or letting them play in the street, compared to his native country, Colombia: “In Colombia, there is a lot of crime. You can’t do anything outdoors in my country. In Spain, you go for a run and you aren’t afraid of being robbed. In my country, the fear of going out is huge” (P3, female, income level V).

### 3.6. Educational Burden

It refers to the burden of homework and school-related activities that children must engage in after the school day. Parents (*n* = 5) narrate how their children have to study a lot, both during school hours and during “so-called” leisure time. The complaint shared by all parents is that the educational burden is greater and more important than enjoying leisure time. The time dedicated to homework is a deterrent for the practice of extra-curricular activities: “They have a great deal of theory. They come super tired (from school), they have to study and they don’t want to do anything else” (P11, male, income level IV).

Most of the interviewees (*n* = 10) felt that the school curriculum does not assign enough importance to physical activities, as the theoretical burden is very demanding: “The excess of homework compels them not to do sports or have fun” (P9, female, income level III); “The education system has bad schedules that do not let the child be a child. If school schedules were better organized, they could have more time to play and be children” (P6, female, income level III).

### 3.7. Community Living

Sometimes, problems arise in the neighborhood communities, because of community norms that do not allow children to play certain activities in the courtyards. Specifically, it is often forbidden to play ball: “In the communities you can’t play, the rules of the communities don’t allow them to play ball, they are children, that’s what they should do” (P9, female, income level III).

Parents (*n* = 4) described how in their countries of origin there is more freedom to play or practice physical activity in the street compared to Spain. They report that, regarding the community relations between neighbors, it is accepted that children should play and do physical activity (sport): “In my country, we don’t live in buildings, we can all play in the street, people go out and everyone starts to play, they go on their bikes or dance. We don’t get angry about these things” (P10, male, income level V).

### 3.8. The Reason for Immigrating

Most parents (*n* = 10) acknowledged that their reason for immigrating was financial. They need to earn money to be able to send it back to their native country, or save enough to return to their country in the future. Thus, physical activity is not a priority for them. This influences their children’s physical activity and the time they spend with them, since the goal is to work more to earn more: “An immigrant wants to return to his or her country. Earn money and return. We come here to work, to earn money, and we don’t have time to be active, sometimes we don’t even have time for our own children” (P7, male, income level IV).

## 4. Discussion

The socio-demographic data shown in Table 1 contextualize the results obtained from the participants’ narratives. Accordingly, in order to understand the results, it is necessary to contextualize which participants provided narrations regarding their experience [27,33]. In this study, the participants were mostly middle-aged women, most of whom did not engage in physical activity in their free time and earned less than 1550 to 2200 euros per month.

This paper aimed to describe the meaning of children’s physical activity from the perspective of immigrant parents. Previous studies [3,4,5,11,24] show how young people and children whose parents are immigrants were less physically active than their native counterparts.

Our results coincide with previous studies [38] showing that physical activity is perceived by parents as a health benefit and should be practiced because of its physical and psychological effects. In addition, our participants perceived that physical activity also has a socializing effect. Similar results have been published by Carratalá and Carratalá [39] and Delgado [40], showing how physical activity is a socializing agent, in terms of integrating rules, as well as developing responsibility and a sense of companionship.

Physical activity is perceived as being very closely related to movement [41,42,43,44]. Our participants reported that in certain physical activities and sports there is a gender distribution. In Spain, the survey of sports habits published by the Ministry of Education, Culture and Sport [45], shows that boys mostly practice sports such as soccer, cycling and swimming, and girls mostly practice gymnastics, hiking and swimming.

In most interviews, participants compared their country of origin with Spain. They used the words “there and here” a lot. There is an important influence between what they knew before and what they know now, with important economic and cultural influences.

Parents find it difficult to be physically active. Previous studies describe how the lower the socio-economic level, the less possibility of physical practice there is [11,46]. Participants in our study acknowledged that the availability of financial resources significantly influences the decisions made regarding their child’s physical activity. Our results highlight that participants prioritize the financial well-being of the family over any health or leisure activity. Bhatnagar et al. [4] describe how this situation tends to change in the following generations, because having access to greater financial resources (a more stable livelihood) allows the family’s priorities to be reorganized. Our results are in line with the qualitative study by Condon and McClean [12], describing that immigrants who are undergoing financial hardships must focus on improving the family economy, being unable to participate or promote the physical activity of their children. Foreigners do not have a socioeconomic level that allows them to justify an added economic expense, since the socioeconomic level of foreigners in Spain is low or medium [47]. According to the 2007 National Immigrant Survey, 40% of foreign nationals work in unskilled labor whereas 22% work in skilled labor [47]. These two categories have the lowest overall purchasing power [26].

There is also a barrier related to infrastructure. The neighborhoods (southern area of Madrid, Spain), where the interviews took place, are not adapted in terms of the number of parks, sports or leisure facilities. The physical environment, with large parks or natural spaces, is a key factor for good physical practice. If the physical (urban) environment often appears as a barrier to the practice of sports, physical and leisure activities, this is exacerbated in the case of immigrant parents, who are used to having a more open and natural physical environment in their countries of origin.

Lack of time is another limiting factor. The parents in this study described having long and overwhelming working hours that prevent them from doing any physical activity. Reimers et al. [8] reported that many parents cannot enroll their children in sports activities, let alone participate in them together. In addition, Reimers et al. [8] identified reduced physical activity related to the difficulty in accessing sports clubs and centers.

The proposed schedules for organized sports activities were considered by some of our participants as an added difficulty, as they described them as incompatible with their work schedules and family life rhythms. Previous studies [24,48,49] state that workloads and family burdens may prevent children and parents from practicing physical activity. In our study, five of the participants had three or more children. A large family could explain the difficulties in reconciling and organizing the family’s daily routine when balancing the different activities, which could have an impact on enrolling their children in extracurricular or leisure activities.

Also, insecurity or fear of going out can influence the decision to take children to leisure areas. Owen et al. [50] and Motl et al. [51] describe insecurity as a barrier to sport. All these barriers coincide with previous studies [24,50,51,52,53] in research carried out in Spain, the United Kingdom and the USA.

The economic expense of certain physical activities (tuition, travel, etc.) could explain a decline in the practice of exercise among children of immigrant parents. In addition, the parent’s financial situation, together with a precarious job situation and long working hours, and the need to earn money and save it, condition the ability to spend money on their child’s physical activity practice. Condon and McClean [12] describe the difficulty of maintaining the physical health of family members, as opposed to the priority of financial health. Participants in the former study were aware of their children’s lack of physical activity, and acknowledged that they did not get enough exercise because of financial hardship, time constraints or feelings of insecurity in some neighborhoods [24]. Our results show how participants prioritize work and economic status over their children’s physical activity. This could be explained by the fact that our participants have been in Spain for less than five years. This means that the parents still have a precarious and/or vulnerable socioeconomic situation and need to find financial stability before they can incorporate the family and their children into other activities.

Finally, the importance of knowing the perspectives of parents regarding physical activity is underlined, as parents can play a crucial role in determining their children’s sedentary behaviors. Two recent studies in Asia [54,55] showed the mutually interdependent relationship between parents’ and children’s physical activity and screen-viewing behaviors, highlighting the impact on children’s health. The results show the potential mechanistic link between parents’ perspectives on physical activities and children’s health outcomes, showing that parents’ screen-viewing behaviors and rules may be associated with their children’s insufficient physical activity levels and overweight problems. In the opinion of the authors, future lines of intervention for pediatric overweight should consider the inclusion of educational and behavioral programs designed for parents, instead of focusing only on children [54].

This study has several limitations. First, we were unable to carry out a homogeneous grouping of immigrant parents by geographically defined areas. Secondly, we did not identify whether the children of the parents were born in Spain or were transferred from their country of origin. The authors believe that this limitation is controlled, since the aim of the study was to describe the perspective of the parents and not that of the children. Additionally, the present study included 12 participants, and 20 interviews have been performed. Previous qualitative studies [27,33,56] have described how the total number of participants included does not depend on a previous calculation of the sample size, rather it is based on the saturation or redundancy of the information obtained in the interviews. Turner-Bowker et al. [57] reported that 92–97% of the saturation can be analyzed after interview numbers 15 and 20. In addition, our results cannot be extrapolated to all immigrant parents who have children, due to the design used. Nonetheless, these results may help professionals understand parents’ experiences, and their difficulties in promoting physical activity and resources for their children. Finally, this study did not perform a cultural comparison between groups of native parents and immigrant parents.

## 5. Conclusions

This study describes the factors related to the physical activity of South American immigrant children from the parents’ perspective. Our results revealed that the immigrant parents who were recruited in this study perceived that their children’s physical activity was relevant and important to their future lives. However, certain barriers exist which limit and prevent the ability for foreign children to exercise, such as income, time availability or extended work shifts.

These results can be used to guide the programming and organization of physical activities in the school and community environment. It is also essential to provide policies to reconcile family and work, so that the lack of time is not a hindrance to the practice of physical activity. It is essential to create policies for financial aid targeted at the most disadvantaged families. In addition, it is necessary to invest in the creation of safe spaces and sports and leisure infrastructures to promote physical activity within the community.

A future line of research would be to carry out a cultural comparison between groups of immigrant and native parents, using a qualitative ethnographic design.

## Figures and Tables

**Table 1:** Sociodemographic characteristics and participant data.

Characteristics	Participants
**Age (y)**	
Mean ± SD ^1^	41 ± 3.97
Range	36–46
**Country of birth (*n*)**	
Argentina	4
Colombia	4
Ecuador	2
Honduras	2
**Years of residence in Spain (y)**	
Mean ± SD	3.17 ± 1.47
Range	1–5
**Children residing at home (n)**	
Means ± SD	2.75 ± 0.97
Range	2–5
**Age of Children (y)**	
Mean ± SD	5.85 ± 2.98
Range	1–12
**Net monthly household income levels (*n*)** **^2^**	
≥3600 euros (level I)	0
2200 € to 3600 € (level II)	0
1550 € to 2200 € (level III)	2
1050 € to 1550 € (level IV)	4
Less than 1050 € (level V)	6
**Leisure time physical activity (*n*)**	
Mother	
Yes	0
No	7
Father	
Yes	4
No	1

^1^ SD = standard deviation; ^2^ Ministry of Health, Consumer Affairs and Social Welfare. National Health Survey. Spain 2017 (ENSE 2017) [2].
